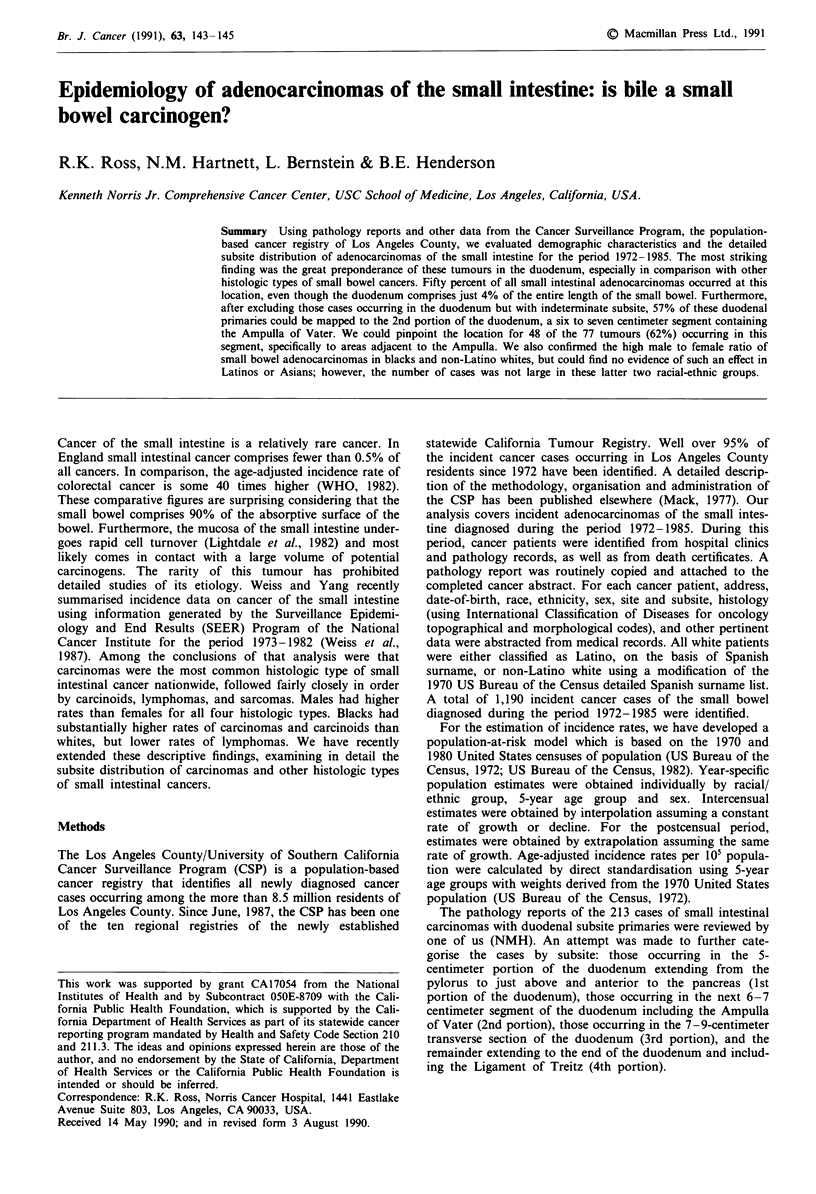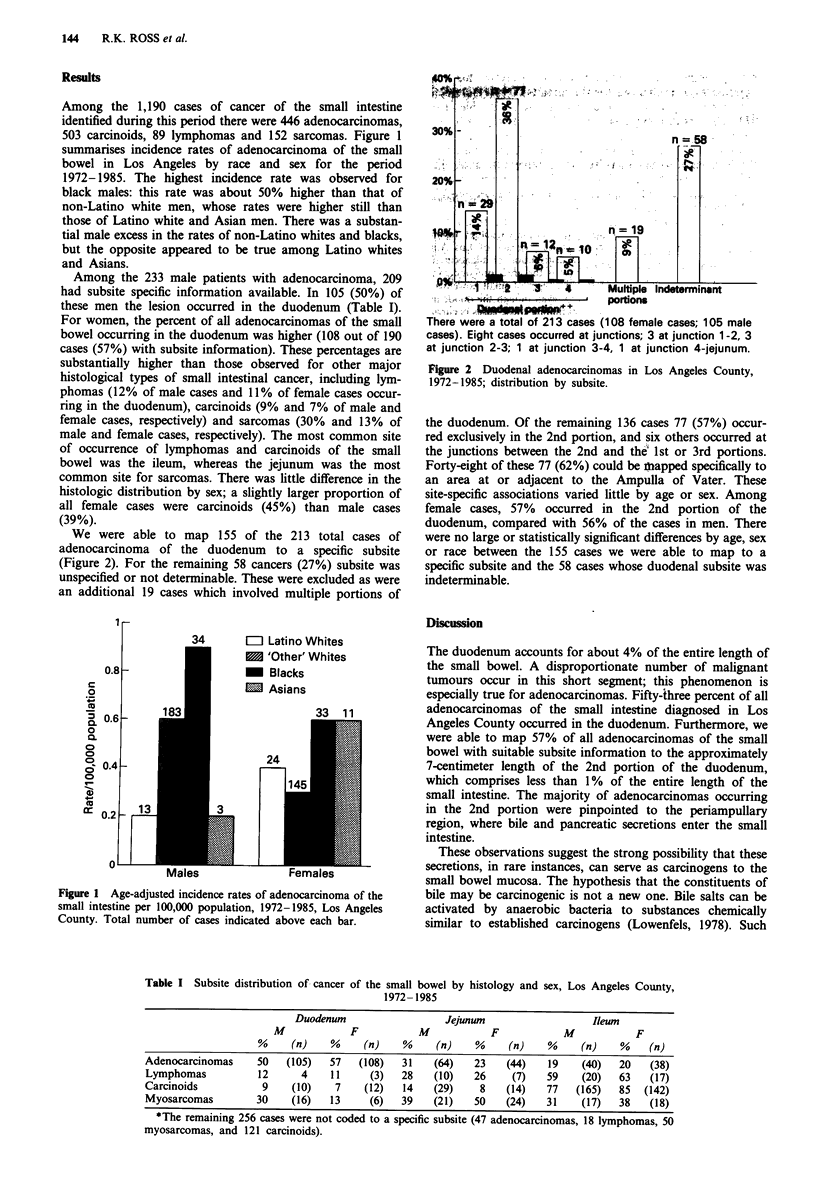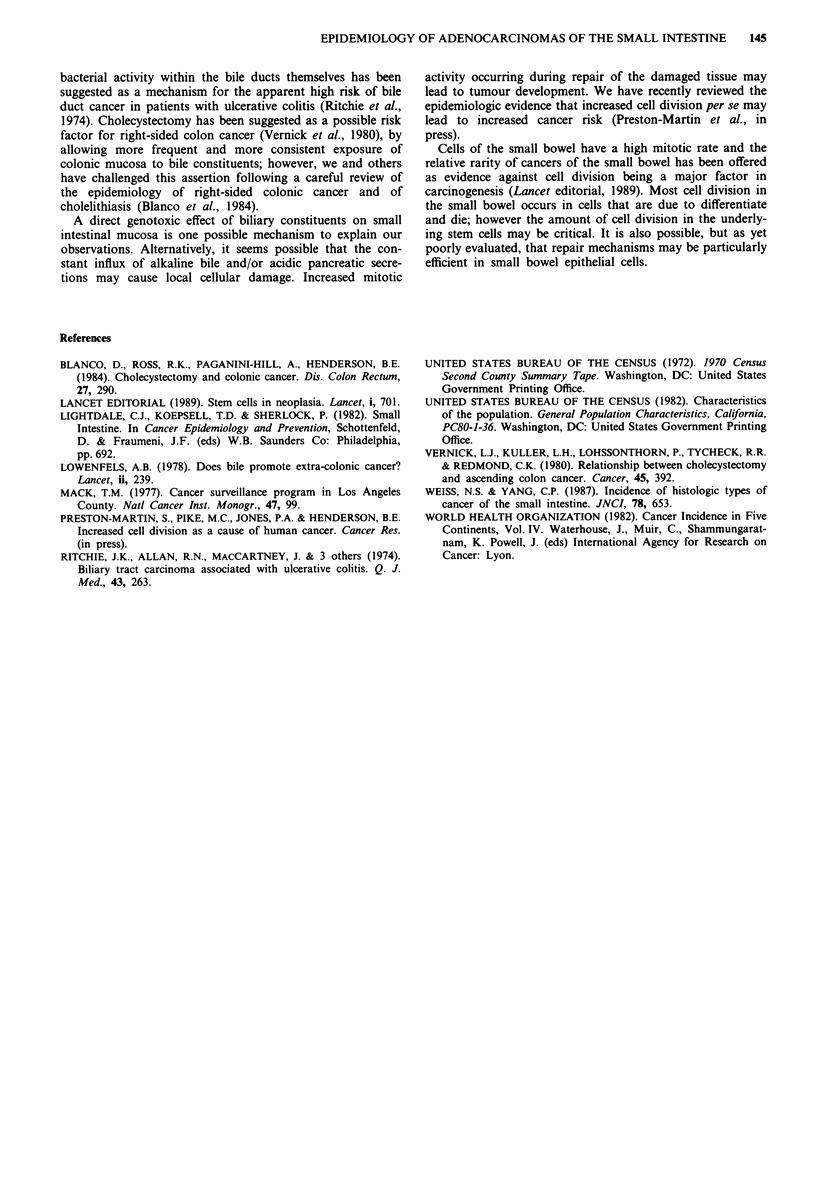# Epidemiology of adenocarcinomas of the small intestine: is bile a small bowel carcinogen?

**DOI:** 10.1038/bjc.1991.29

**Published:** 1991-01

**Authors:** R. K. Ross, N. M. Hartnett, L. Bernstein, B. E. Henderson

**Affiliations:** Kenneth Norris Jr. Comprehensive Cancer Center, USC School of Medicine, Los Angeles.

## Abstract

Using pathology reports and other data from the Cancer Surveillance Program, the population-based cancer registry of Los Angeles County, we evaluated demographic characteristics and the detailed subsite distribution of adenocarcinomas of the small intestine for the period 1972-1985. The most striking finding was the great preponderance of these tumours in the duodenum, especially in comparison with other histologic types of small bowel cancers. Fifty percent of all small intestinal adenocarcinomas occurred at this location, even though the duodenum comprises just 4% of the entire length of the small bowel. Furthermore, after excluding those cases occurring in the duodenum but with indeterminate subsite, 57% of these duodenal primaries could be mapped to the 2nd portion of the duodenum, a six to seven centimeter segment containing the Ampulla of Vater. We could pinpoint the location for 48 of the 77 tumours (62%) occurring in this segment, specifically to areas adjacent to the Ampulla. We also confirmed the high male to female ratio of small bowel adenocarcinomas in blacks and non-Latino whites, but could find no evidence of such an effect in Latinos or Asians; however, the number of cases was not large in these latter two racial-ethnic groups.


Br. J. Cancer (1991), 63, 143-145 © Macmillan Press Ltd., 1991

### Epidemiology of adenocarcinomas of the small intestine: is bile a small bowel carcinogen?

R.K. Ross, N.M. Hartnett, L. Bernstein & B.E. Henderson

*Kenneth Norris Jr. Comprehensive Cancer Center, USC School of Medicine, Los Angeles, California, USA.*

**Summary** Using pathology reports and other data from the Cancer Surveillance Program, the population-based cancer registry of Los Angeles County, we evaluated demographic characteristics and the detailed subsite distribution of adenocarcinomas of the small intestine for the period 1972-1985. The most striking finding was the great preponderance of these tumours in the duodenum, especially in comparison with other histologic types of small bowel cancers. Fifty percent of all small intestinal adenocarcinomas occurred at this location, even though the duodenum comprises just 4% of the entire length of the small bowel. Furthermore, after excluding those cases occurring in the duodenum but with indeterminate subsite, 57% of these duodenal primaries could be mapped to the 2nd portion of the duodenum, a six to seven centimeter segment containing the Ampulla of Vater. We could pinpoint the location for 48 of the 77 tumours (62%) occurring in this segment, specifically to areas adjacent to the Ampulla. We also confirmed the high male to female ratio of small bowel adenocarcinomas in blacks and non-Latino whites, but could find no evidence of such an effect in Latinos or Asians; however, the number of cases was not large in these latter two racial-ethnic groups.

Cancer of the small intestine is a relatively rare cancer. In England small intestinal cancer comprises fewer than 0.5% of all cancers. In comparison, the age-adjusted incidence rate of colorectal cancer is some 40 times higher (WHO, 1982). These comparative figures are surprising considering that the small bowel comprises 90% of the absorptive surface of the bowel. Furthermore, the mucosa of the small intestine undergoes rapid cell turnover (Lightdale *et al.*, 1982) and most likely comes in contact with a large volume of potential carcinogens. The rarity of this tumour has prohibited detailed studies of its etiology. Weiss and Yang recently summarised incidence data on cancer of the small intestine using information generated by the Surveillance Epidemiology and End Results (SEER) Program of the National Cancer Institute for the period 1973-1982 (Weiss *et al.*, 1987). Among the conclusions of that analysis were that carcinomas were the most common histologic type of small intestinal cancer nationwide, followed fairly closely in order by carcinoids, lymphomas, and sarcomas. Males had higher rates than females for all four histologic types. Blacks had substantially higher rates of carcinomas and carcinoids than whites, but lower rates of lymphomas. We have recently extended these descriptive findings, examining in detail the subsite distribution of carcinomas and other histologic types of small intestinal cancers.

### Methods

The Los Angeles County/University of Southern California Cancer Surveillance Program (CSP) is a population-based cancer registry that identifies all newly diagnosed cancer cases occurring among the more than 8.5 million residents of Los Angeles County. Since June, 1987, the CSP has been one of the ten regional registries of the newly established statewide California Tumour Registry. Well over 95% of the incident cancer cases occurring in Los Angeles County residents since 1972 have been identified. A detailed description of the methodology, organisation and administration of the CSP has been published elsewhere (Mack, 1977). Our analysis covers incident adenocarcinomas of the small intestine diagnosed during the period 1972-1985. During this period, cancer patients were identified from hospital clinics and pathology records, as well as from death certificates. A pathology report was routinely copied and attached to the completed cancer abstract. For each cancer patient, address, date-of-birth, race, ethnicity, sex, site and subsite, histology (using International Classification of Diseases for oncology topographical and morphological codes), and other pertinent data were abstracted from medical records. All white patients were either classified as Latino, on the basis of Spanish surname, or non-Latino white using a modification of the 1970 US Bureau of the Census detailed Spanish surname list. A total of 1,190 incident cancer cases of the small bowel diagnosed during the period 1972-1985 were identified.

For the estimation of incidence rates, we have developed a population-at-risk model which is based on the 1970 and 1980 United States censuses of population (US Bureau of the Census, 1972; US Bureau of the Census, 1982). Year-specific population estimates were obtained individually by racial/ethnic group, 5-year age group and sex. Intercensual estimates were obtained by interpolation assuming a constant rate of growth or decline. For the postcensual period, estimates were obtained by extrapolation assuming the same rate of growth. Age-adjusted incidence rates per $10^5$ population were calculated by direct standardisation using 5-year age groups with weights derived from the 1970 United States population (US Bureau of the Census, 1972).

The pathology reports of the 213 cases of small intestinal carcinomas with duodenal subsite primaries were reviewed by one of us (NMH). An attempt was made to further categorise the cases by subsite: those occurring in the 5-centimeter portion of the duodenum extending from the pylorus to just above and anterior to the pancreas (1st portion of the duodenum), those occurring in the next 6-7 centimeter segment of the duodenum including the Ampulla of Vater (2nd portion), those occurring in the 7-9-centimeter transverse section of the duodenum (3rd portion), and the remainder extending to the end of the duodenum and including the Ligament of Treitz (4th portion).

This work was supported by grant CA17054 from the National Institutes of Health and by Subcontract 050E-8709 with the California Public Health Foundation, which is supported by the California Department of Health Services as part of its statewide cancer reporting program mandated by Health and Safety Code Section 210 and 211.3. The ideas and opinions expressed herein are those of the author, and no endorsement by the State of California, Department of Health Services or the California Public Health Foundation is intended or should be inferred.
Correspondence: R.K. Ross, Norris Cancer Hospital, 1441 Eastlake Avenue Suite 803, Los Angeles, CA 90033, USA.
Received 14 May 1990; and in revised form 3 August 1990.

### Results

Among the 1,190 cases of cancer of the small intestine identified during this period there were 446 adenocarcinomas, 503 carcinoids, 89 lymphomas and 152 sarcomas. Figure 1 summarises incidence rates of adenocarcinoma of the small bowel in Los Angeles by race and sex for the period 1972–1985. The highest incidence rate was observed for black males: this rate was about 50% higher than that of non-Latino white men, whose rates were higher still than those of Latino white and Asian men. There was a substantial male excess in the rates of non-Latino whites and blacks, but the opposite appeared to be true among Latino whites and Asians.

Among the 233 male patients with adenocarcinoma, 209 had subsite specific information available. In 105 (50%) of these men the lesion occurred in the duodenum (Table I). For women, the percent of all adenocarcinomas of the small bowel occurring in the duodenum was higher (108 out of 190 cases (57%) with subsite information). These percentages are substantially higher than those observed for other major histological types of small intestinal cancer, including lymphomas (12% of male cases and 11% of female cases occurring in the duodenum), carcinoids (9% and 7% of male and female cases, respectively) and sarcomas (30% and 13% of male and female cases, respectively). The most common site of occurrence of lymphomas and carcinoids of the small bowel was the ileum, whereas the jejunum was the most common site for sarcomas. There was little difference in the histologic distribution by sex; a slightly larger proportion of all female cases were carcinoids (45%) than male cases (39%).

We were able to map 155 of the 213 total cases of adenocarcinoma of the duodenum to a specific subsite (Figure 2). For the remaining 58 cancers (27%) subsite was unspecified or not determinable. These were excluded as were an additional 19 cases which involved multiple portions of the duodenum. Of the remaining 136 cases 77 (57%) occurred exclusively in the 2nd portion, and six others occurred at the junctions between the 2nd and the 1st or 3rd portions. Forty-eight of these 77 (62%) could be mapped specifically to an area at or adjacent to the Ampulla of Vater. These site-specific associations varied little by age or sex. Among female cases, 57% occurred in the 2nd portion of the duodenum, compared with 56% of the cases in men. There were no large or statistically significant differences by age, sex or race between the 155 cases we were able to map to a specific subsite and the 58 cases whose duodenal subsite was indeterminable.

**Figure 1** Age-adjusted incidence rates of adenocarcinoma of the small intestine per 100,000 population, 1972–1985, Los Angeles County. Total number of cases indicated above each bar.

There were a total of 213 cases (108 female cases; 105 male cases). Eight cases occurred at junctions; 3 at junction 1-2, 3 at junction 2-3; 1 at junction 3-4, 1 at junction 4-jejunum.

**Figure 2** Duodenal adenocarcinomas in Los Angeles County, 1972–1985; distribution by subsite.

### Discussion

The duodenum accounts for about 4% of the entire length of the small bowel. A disproportionate number of malignant tumours occur in this short segment; this phenomenon is especially true for adenocarcinomas. Fifty-three percent of all adenocarcinomas of the small intestine diagnosed in Los Angeles County occurred in the duodenum. Furthermore, we were able to map 57% of all adenocarcinomas of the small bowel with suitable subsite information to the approximately 7-centimeter length of the 2nd portion of the duodenum, which comprises less than 1% of the entire length of the small intestine. The majority of adenocarcinomas occurring in the 2nd portion were pinpointed to the periampullary region, where bile and pancreatic secretions enter the small intestine.

These observations suggest the strong possibility that these secretions, in rare instances, can serve as carcinogens to the small bowel mucosa. The hypothesis that the constituents of bile may be carcinogenic is not a new one. Bile salts can be activated by anaerobic bacteria to substances chemically similar to established carcinogens (Lowenfels, 1978). Such

**Table I** Subsite distribution of cancer of the small bowel by histology and sex, Los Angeles County, 1972–1985

| | *Duodenum* | | | | *Jejunum* | | | | *Ileum* | | | |
|---|---|---|---|---|---|---|---|---|---|---|---|---|
| | *M* | | *F* | | *M* | | *F* | | *M* | | *F* | |
| | % | (n) | % | (n) | % | (n) | % | (n) | % | (n) | % | (n) |
| Adenocarcinomas | 50 | (105) | 57 | (108) | 31 | (64) | 23 | (44) | 19 | (40) | 20 | (38) |
| Lymphomas | 12 | 4 | 11 | (3) | 28 | (10) | 26 | (7) | 59 | (20) | 63 | (17) |
| Carcinoids | 9 | (10) | 7 | (12) | 14 | (29) | 8 | (14) | 77 | (165) | 85 | (142) |
| Myosarcomas | 30 | (16) | 13 | (6) | 39 | (21) | 50 | (24) | 31 | (17) | 38 | (18) |

*The remaining 256 cases were not coded to a specific subsite (47 adenocarcinomas, 18 lymphomas, 50 myosarcomas, and 121 carcinoids).

bacterial activity within the bile ducts themselves has been suggested as a mechanism for the apparent high risk of bile duct cancer in patients with ulcerative colitis (Ritchie *et al.*, 1974). Cholecystectomy has been suggested as a possible risk factor for right-sided colon cancer (Vernick *et al.*, 1980), by allowing more frequent and more consistent exposure of colonic mucosa to bile constituents; however, we and others have challenged this assertion following a careful review of the epidemiology of right-sided colonic cancer and of cholelithiasis (Blanco *et al.*, 1984).

A direct genotoxic effect of biliary constituents on small intestinal mucosa is one possible mechanism to explain our observations. Alternatively, it seems possible that the constant influx of alkaline bile and/or acidic pancreatic secretions may cause local cellular damage. Increased mitotic activity occurring during repair of the damaged tissue may lead to tumour development. We have recently reviewed the epidemiologic evidence that increased cell division *per se* may lead to increased cancer risk (Preston-Martin *et al.*, in press).

Cells of the small bowel have a high mitotic rate and the relative rarity of cancers of the small bowel has been offered as evidence against cell division being a major factor in carcinogenesis (*Lancet* editorial, 1989). Most cell division in the small bowel occurs in cells that are due to differentiate and die; however the amount of cell division in the underlying stem cells may be critical. It is also possible, but as yet poorly evaluated, that repair mechanisms may be particularly efficient in small bowel epithelial cells.

**References**

BLANCO, D., ROSS, R.K., PAGANINI-HILL, A., HENDERSON, B.E. (1984). Cholecystectomy and colonic cancer. *Dis. Colon Rectum*, **27**, 290.

LANCET EDITORIAL (1989). Stem cells in neoplasia. *Lancet*, **i**, 701.

LIGHTDALE, C.J., KOEPSELL, T.D. & SHERLOCK, P. (1982). Small Intestine. In *Cancer Epidemiology and Prevention*, Schottenfeld, D. & Fraumeni, J.F. (eds) W.B. Saunders Co: Philadelphia, pp. 692.

LOWENFELS, A.B. (1978). Does bile promote extra-colonic cancer? *Lancet*, **ii**, 239.

MACK, T.M. (1977). Cancer surveillance program in Los Angeles County. *Natl Cancer Inst. Monogr.*, **47**, 99.

PRESTON-MARTIN, S., PIKE, M.C., JONES, P.A. & HENDERSON, B.E. Increased cell division as a cause of human cancer. *Cancer Res.* (in press).

RITCHIE, J.K., ALLAN, R.N., MACCARTNEY, J. & 3 others (1974). Biliary tract carcinoma associated with ulcerative colitis. *Q. J. Med.*, **43**, 263.

UNITED STATES BUREAU OF THE CENSUS (1972). *1970 Census Second County Summary Tape*. Washington, DC: United States Government Printing Office.

UNITED STATES BUREAU OF THE CENSUS (1982). Characteristics of the population. *General Population Characteristics, California, PC80-1-36*. Washington, DC: United States Government Printing Office.

VERNICK, L.J., KULLER, L.H., LOHSSONTHORN, P., TYCHECK, R.R. & REDMOND, C.K. (1980). Relationship between cholecystectomy and ascending colon cancer. *Cancer*, **45**, 392.

WEISS, N.S. & YANG, C.P. (1987). Incidence of histologic types of cancer of the small intestine. *JNCI*, **78**, 653.

WORLD HEALTH ORGANIZATION (1982). Cancer Incidence in Five Continents, Vol. IV. Waterhouse, J., Muir, C., Shammungaratnam, K. Powell, J. (eds) International Agency for Research on Cancer: Lyon.